# Mohawk protects against tendon damage via suppressing Wnt/β-catenin pathway

**DOI:** 10.1016/j.heliyon.2024.e25658

**Published:** 2024-02-06

**Authors:** Ziming Liu, Wenfeng Han, Jiao Meng, Yanbing Pi, Tong Wu, Yifei Fan, Qinwei Guo, Xiaoqing Hu, Yuhua Chen, Wenxiao Jiang, Feng Zhao

**Affiliations:** a Department of Sports Medicine, Sports Medicine Institute, Beijing Key Laboratory of Sports Injuries, Peking University Third Hospital, Beijing, China; b Engineering Research Center of Sports Trauma Treatment Technology and Devices, Ministry of Education, Peking University Third Hospital, Beijing, China; c Department of Orthopedics, General Hospital of Northern Theater Command, Shenyang, Liaoning, China; d Department of Neurosurgery, Bijie Traditional Chinese Medical Hospital, Bijie, Guizhou, China; e Department of Sports Medicine, Qilu Hospital of Shandong University (Qingdao Campus), Qingdao, Shandong, China

**Keywords:** Tendon damage, Mkx, Tension strength, Wnt/β-catenin, Mechanical force

## Abstract

Degenerative tendon injuries are common clinical problems associated with overuse or aging, and understanding the mechanisms of tendon injury and regeneration can contribute to the study of tendon healing and repair. As a transcription factor, Mohawk (Mkx) is responsible for tendons development, yet, the roles of which in tendon damage remain mostly elusive. In this study, using Mkx overexpressed mice on long treadmill as an in vivo model and Mkx^OE^ Achilles tenocytes stimulated by equiaxial stretch as an in vitro model, we anaylsed the effects of Mkx overexpression on the tendon. Mkx and tendon tension strength were decreased after the expose to excessive mechanical forces, and Mkx overexpression protected the tendon from damage. Moreover, we revealed that the Wnt/β-catenin activation, inflammation, and Runx2 expression were increased at the injured Achilles tendon, upregulated Mkx significantly reversed the increased Wnt/β-catenin pathway, Tnf-α, Il-1β, and Il-6 levels, and reduced tendon cell damage. However, Wnt3a, IWR and BIO had not significantly affected the Mkx expression in achilles tenocytes. In conclusion, Mkx is involved in tendon healing and protects the tendon from damage through suppressing Wnt/β-catenin pathway, suggesting Mkx/Wnt/β-catenin pathway may be potential therapeutic targets for tendon damage.

## Highlights

• Mkx and tendon tension strength were decreased after the exposure of excessive mechanical forces.
• Mkx overexpression protected the tendon from damage.
• Up-regulated Mkx significantly reversed increased Wnt/β-catenin signaling, inflammation, and tendon cell damage.
• Wnt pathway had non significantly affect the Mkx expression in achilles tenocytes.

## 1 Introduction

Tendons play a vital role in musculoskeletal system, which is subjected to a great deal of load and mechanical stress in our daily life and exercise, transferring mechanical load and enabling movement [1,2]. Tendon injury can result from prolonged exercise, inappropriate training, lack of muscle strength, or muscle imbalance, so that is a common clinical problem and is often difficult to fully recover [3]. It may be due to its special tissue type, few blood vessels and cells [4], and still face the possibility of re-fracture after surgery [5], coupled with limited understanding of it, leading to some treatment delays, resulting in unsatisfactory clinical treatment results [6,7]. Therefore, understanding tendon injury and regeneration mechanisms can contribute to the study of tendon healing and repair.

Tendons are responsible for stretching and contracting muscles and mainly composed of glial proteins, including types I, III, IV, V, and VI collagen, proteoglycans (firegulin [Fmod], hyperglycans, decorin, and lumican), and glycoproteins (elastin, fibrin, and tendinoproteins), etc., while type I collagen is essential for its elasticity [[8], [9], [10]]. Recently, transcription factors Scleraxis (Scx) and Mohawk (Mkx) have been considered essential transcription factors for tendon development [[11], [12], [13], [14]]. Scx is expressed in tendon progenitor cells and all tendon tissue cells [15,16]. Mkx as a TALE-family homeodomain, is essential for a large set of both developmental and maturation into adulthood, including cell proliferation, differentiation, and positional specification [17,18]. In mesenchymal stem cells (MSCs), Mkx overexpression improves tendon-related markers and promotes the diameter of tendon's collagen fibers [19]. Mkx inhibits angiofibrosis by suppressing tendon stem/progenitor cells differentiation into myofibroblasts [20]. While Mkx knockout (KO) mice showed, Mkx deficiency leads to a decrease of collagen fibers that leads to general tendon hypoplasia through downstream genes of Col1a1 gene and proteoglycans, which regulates fiber growth [13,21,22].

The Wnt/β-catenin pathway is involved in bone development and the adaptive response of bone to physical stimuli [23,24], mechanical loading modulates Wnt signaling in osteocytes, through Wnt-3a, Wnt5a, LRP-5, LRP-6 and β-catenin [25,26]. Tendons are load-bearing organs, and it has been reported that tendon injury alters Scx, Fmod, MMP2 and Col1 expression [[27], [28], [29]]. Despite the long-term expression of Mkx after the developmental stage, and its role in tendon homeostasis, there are still rare researches linking Mkx to tendon injuries. The analysis results of Mkx KO mice provide important information for tendon development, but it is not clear what the change of MKX in the process of tendon injury and post-injury, and relevance of Mkx and Wnt signaling pathway remains unsharpness. We hypothesize that Mkx plays an important role in the repair and regeneration of tendon injuries, which relates to activation of Wnt signaling. This work is to analysis the role of Mkx in tendon injuries, and explore the relationship of Mkx and Wnt signaling.

## 2 Methods

### 2.1 Treadmill

All animal experiments were conducted in accordance with the program approved by the Institutional Animal Care and Use Committee at the Peking University. The Mkx^OE^ (Mkx overexpressed) and wide type (WT) C57BL/6 mice were generated and analyzed at Peking University Laboratory Animal Center. The 12-week-old animals were placed on a treadmill system (ZSDB, Beijing, China) and exercised according to the assigned treadmill regimen. The exercise consistsed of 15 m/min of horizontal exercise, 60 min at a time, five days a week, for 12 weeks after a period of acclimatization. Control group free cage activity.

### 2.2 Primary mice tenocytes cultivation

The 6-week-old mice were euthanized and immersed in 70% ethanol for 30s. After the Achilles tendon is removed, the Achilles tendon is cut into 1mm3 pieces and digested by trypsin (Gibco) for 30 min and by collagenase (Roche) for 45 min. The dissolved tissues were filtered and cultured with DMEM medium with 20% FBS and 1% penicillin-streptomycin, at 37 °C in 5% CO_2_. After cultured for 5–7 days, the medium was replaced to DMEM plus 10% FBS, and normal passage and cell experiments were carried out.

### 2.3 Cell stretching

Achilles tenocytes were stretched using Flexcell® FX-5000™ Tension System (Flexcell International). After trypsinized and seeded onto a type I-collagen-coated chamber (BioFLEX® Cultrue Plates) and incubated until cells attached, cells were stretched at various stretch magnitudes (1%, 2%, 4%, 8% and 12%) and frequencies (1.0 Hz) for 4 h, at 37 °C in 5% CO_2_.

### 2.4 Tensile testing

We used the entire Achilles tendon unit to evaluate the mechanical properties of the Achilles tendon, using the uniaxial material test system (Autograph AGS-G; Shimadzu Corp. Ltd.) with a 500 N load cell to measure tensile properties. In order to facilitate grasping during the experiment, the proximal end of the Achilles tendon and the foot of the mice were fixed in a special clamp, and the specimens were pulled at a constant strain rate of 0.5 mm/s. All samples were broken within gauge length. Force data are collected at a frequency of 50 Hz in Trapezium (Shimadzu Corp. Ltd.) software. For each specimen, the stress-strain curve is established from the load-displacement curve, and the Young's modulus of each stress-strain curve is calculated using the cross-sectional area.

### 2.5 Transient transfection

The primary achilles tenocytes were respectively transfected with 1 μg Mkx mice cDNA ORF Clone (Lenti ORF clone of Mkx, Origene, MR225168L2) according to the manufacturer's instructions (Origene, Beijing). After 48 h infection, the Mkx mRNA overexpression was determined by qRT-PCR.

### 2.6 Quantitative real-time PCR

Total RNA of Achilles tendon tissue and primary Achilles tenocytes were lysed in Trizol reagent (Thermo Scientific, Wilmington, USA) and transcribed in reverse to cDNA by HiFi-MMLV cDNA first strand synthesis Kit (CW Bio, Beijing, China). The GoTaq qPCR Master Mix (Promega) was conducted to quantitative real-time PCR by CFX96TM Real-Time System (Bio-Rad). GAPDH was selected for internal control. The list of primer sequences for RT-PCR is shown in Table 1.

**Table 1:** Primers for RT-qPCR.

Primer	Forward 5′–3′	Reverse 5′–3′
Acan	CTGTCTATCTGCACGCCAACC	CCTCTTCACCACCCACTCCGA
Alpl	GCACAACATCAAGGACATCG	TCAGTTCTGTTCTTGGGGTACAT
Col1a1	GTCCGAGGTCCTAATGGAGATGC	GGTCCAGGGAATCCGATGT
Col2a1	CCAGGTCCTGCTGGAAAA	CCTCTTTCTCCGGCCTTT
Dcn	GACTCCACGACAATGAGATCACC	GTTGCCATCCAGATGCAGTTC
Fmod	CAAGGCAACAGGATCAATGAG	CTGCAGCTTGGAGAAGTTCA
Mkx	GCTCCGGACAGCTTCTCCTATTG	CTGGGTGAGCCTAGGGTTCAG
Gapdh	GGCAAGTTCAATGGCACAGT	TGGTGAAGACGCCAGTAGACTC
Runx2	CCACAGAGCTATTAAAGTGACAGTG	AACAAACTAGGTTTAGAGTCATCAAGC
Osx	TTCTTCACTGTGGGGCAAC	GCACCAGGATACACAACACCT
Tnmd	CTACAGCAATGGCGAGAAGAAGAAG	GACCTACAAAGTAGATGCCAGTGTATC
Wnt3	TGGCTTCAGCATCTGTTACCTTC	AAGATC CCCATACTGCCATCAC
Wnt5	CTTCGCCCGGGAGTTTGTGGA	CGGCGGCGCTGTCGTATTTC
β-catenin	CAGAAGCTATTGAAGCTGAGG	TTCCATCATGGGGTCCATAC

### 2.7 CCK8 assay

Cell death was assessed by CCK8 assay. After stretching, Achilles tenocytes were added with 10 μL CCK8 solution (Dojindo Laboratories, Tokyo, Japan) and incubated at 37 °C for 4 h, then microplate reader (Thermo Scientific) was conducted to detect the OD450.

### 2.8 LDH release assessment

60, 000/ml Achilles tenocytes were plated into 96-well plates with 100 μl/well and then underwent stretching and treated with different agents according to the different experimental groups. After treatment, LDH was measured according to the LDH Cytotoxicity Assay Kit instructions (Cat: C0016; Beyotime Biotechnology, Shanghai, China). Finally, absorbance at 490 nm was recorded.

### 2.9 Inflammatory factors analysis

After collected the tissues lysis and Achilles tenocytes culture medium supernatant, protein concentration was assessed by BCA assay kit (Thermo Scientific), and ELISA analysis of Tnf-α, Il-1β, and Il-6 were performed, according to the manufacturer's instructions (Beyotime, Shanghai, China).

### 2.10 Western blotting

After collected cell extract, the protein samples were separated in SDS-PAGE running buffer, and transferred onto polyvinylidene difluoride membrane. After isolation for 1

the corresponding primary antibody was incubated at 4 °C overnight: Anti-Mkx (1/200, Abcam), Anti-Tnmd (1/200, Abcam), Anti-Col1a1 (1/1000, Abcam), Anti-Osx (1/1000, Abcam), Anti-Col2a1 (1/2000, Abcam), Anti-Runx2 (1/1000, Abcam), Anti-Wnt3a (1/1000, Abcam), Anti-Wnt5a (1/1000, Abcam) and Anti-β-catenin (1/10,000, Abcam), and Anti-GAPDH antibody (1/20,000, Abcam), then incubated with horseradish peroxidase (HRP)-conjugated antibodies (Abgent, ASS3403, 1/20,000). The signal was revealed with an ECL-Detection Kit (Millipore, USA).

### 2.11 Immunofluorescence staining

After treatment, Achilles tenocytes were fixed with 4% paraformaldehyde for 30 min and permeabilized with 0.1% Triton-X for 10 min, then blocked and incubated with the β-catenin primary antibody (1/100, Abcam). Fluorescence-conjugated secondary antibodies was incubated at room temperature for 1 h away from light and cell nuclei was stained by Hoechst 33,258. Images were obtained by a fluorescence microscope (Leica, Oskar-Barnack, Germany).

### 2.12 Statistical analysis

The results are expressed as mean ± SD. All statistical analyses were conducted by SPSS statistical software (version 22.0, IBM, Armonk, NY, USA). To compare differences between two groups, normally distributed continuous variables were compared by Student's *t*-test. For multiple comparisons more than two groups, data were analyzed using one-way analysis of variance (ANOVA) followed by Tukey-Kramer post-hoc test. *P < 0.05 was considered significant*.

## 3 Results

### 3.1 Long term mechanical loading reduced Mkx expression as well as achilles tendon tension strength

**Fig. 1:**
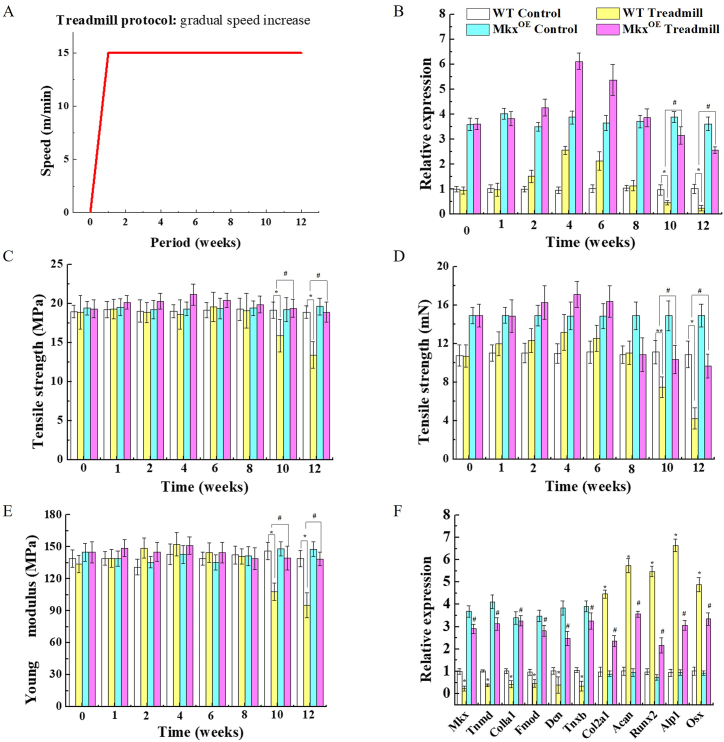
Excessive mechanical loading reduced Mkx and Achilles tendon tension strength in vivo. (A) A schematic illustrating the specific treadmill protocol. A control mice was allowed to move freely within the cage. (B, C, D and E) Mkx gene expression and Achilles tendon tension strength following treadmill exercise. (B) Mkx of Achilles tendon was significantly elevated following initial treadmill exercise (0∼6w), but significantly reduced when sustain excessive mechanical loading (10∼12w). (C, D and E) Tensile strength, tensile strength per unit area and Young's modulus of tendon were detected after treadmill exercise. Excessive mechanical loading reduced tendon strength, while tendon strength maintained a good trend in Mkx^OE^ mice. (F) Tendon-associated gene expression following excessive treadmill exercise. Mkx, Tnmd, Col1a1, Fmod, Dcn, and Tnxb were significantly reduced following treadmill exercise. Treadmill exercise in Mkx^OE^ mice results in either no change or only a marginal increase in tendon-associated genes. Nevertheless, osteogenesis- and chondrogenesis-related genes Col2a1, Acan, Runx2, Alpl, and Osx, had the opposite trend. Error bars represent standard errors of the means (n = 5) (*, *P* < 0.05, *vs.* WT control; #, *P* < 0.05, *vs.* WT treadmill; Tukey-Kramer post-hoc test).

The influence of mechanical load on a mouse treadmill model was examined, through temperate treadmill exercise for 12 weeks after one week of acclimatization (Fig. 1A). We observed Mkx mRNA in a range of time points (0/1/2/4/6/8/10/12 weeks) following mechanical loading (Fig. 1B). Moderate mechanical loading resulted in a significant increase in Mkx during the initial stage (0–4 weeks), followed by a gradual decrease during 4–12 weeks, resulting a peak expression of Mkx at 4 weeks. The trend of Achilles tendon tension strength was similar to the pattern of Mkx mRNA level. Mechanical stimulation significantly increased the tensile strength, tensile strength per unit area and Young's modulus of tendon in a period of 4 weeks, tendon tension strength progressively decreased with the subsequent loading (Fig. 1C, D, E). The tendon-related genes, including Tnmd, Col1a1, Fmod, decorin (Dcn), and tenascin XB (Tnxb) were marginal in Achilles tendons at 12 w (Fig. 1F). Osteogenesis- and chondrogenesis-related markers, including Col2a1, Acan, Runx2, Alpl, and Osx, were increased at 12 w (Fig. 1F). The correlation of Mkx expression and tendon damage suggests Mkx may play a role in tendon damage. To investigate Mkx function in tendon damage, we employed Mkx overexpression mice model. After the same exercise was enforced in Mkx^OE^ mice, Mkx mRNA still tended to increase first and then decrease later like the WT mice (Fig. 1B), and there was no obvious alteration in tendon tension, still maintained high expression of tendon-related markers, and a much less increase in osteogenesis- and chondrogenesis-related gene levels at 12w, compared with WT control mice (Fig. 1C ∼ F).

### 3.2 Mkx protect tendon through suppressing Wnt/β-catenin pathway

**Fig. 2:**
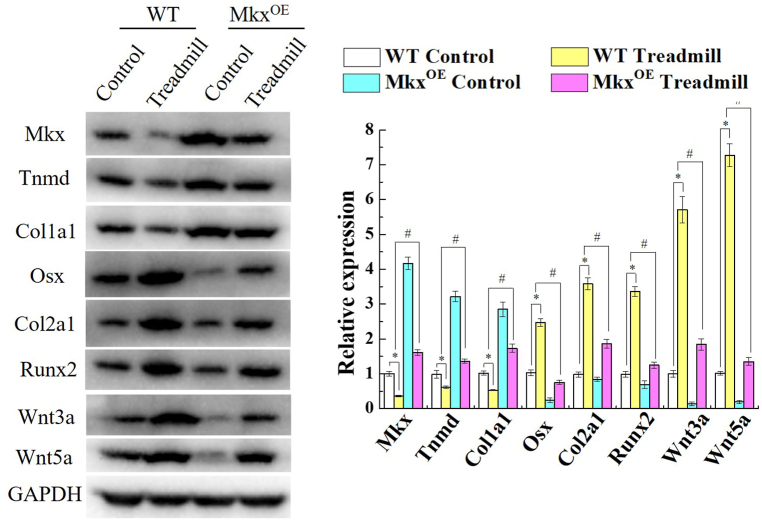
Mkx reduced Wnt/β-catenin activation in Achilles tendon. Relative expressions of Mkx, Col1a1, Tnmd, Col2a1, Runx2, Osx, Wnt3a and Wnt5a in WT and Mkx^OE^ Achilles tendon tissues, were detected by WB after treadmill exercise at 12 weeks. The full uncropped gels and blots images were shown in Fig. S1. Mean and SD are indicated (n = 5) (*, *P* < 0.05, *vs.* WT control; #, *P* < 0.05, *vs*. WT treadmill; Tukey-Kramer post-hoc test).

**Fig. 3:**
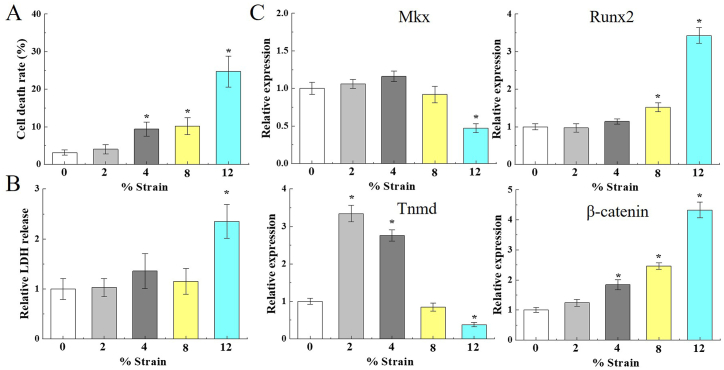
Excessive mechanical stretching reduces tenocytes activity and Mkx in primary mice achilles tenocytes in vitro. Primary mice achilles tenocytes were seeded onto collagen-coated chambers and respectively undergone to 2%, 4%, 8% and 12% strain, 1.0 Hz frequency equiaxial stretch for 4 h, and then detected cell viability by CCK8 assay (A) and LDH release (B). (C) Mkx and Tnmd mRNA were reduced, while Runx2 and β-catenin mRNA were elevated because of tenocyte stretching under 12% strain. Mean and SD are indicated (n = 5) (*, *P* < 0.05, *vs.* 0% strain; Tukey-Kramer post-hoc test).

Mkx expression was detected by WB in Achilles tendon tissues, after treadmill exercise. WB results showed that (Fig. 2), Mkx and tendon-related markers, Col1a1 and Tnmd, were evidently lower in WT treadmill mice, compared with WT control mice. Conversely, Wnt3a, Wnt5a, Col2a1, Runx2 and Osx were obviously high expressions in Achilles tendon of WT treadmill mice. When Mkx was overexpressioned, Wnt3a, Wnt5a, Col2a1, Runx2 and Osx were conspicuously suppressed in Achilles tendon tissue of Mkx^OE^ mice, and Col1a1 and Tnmd were maintained by Mkx. Results showed that long term exercise load causes deficiency of tendon-related markers, while Mkx can reverse this. In vitro study, tenocytes were subjected to 2%, 4%, 8% and 12% strain, with 1.0 Hz frequency equiaxial stretch for 4 h, and cytoactive was measured through CCK8 assay and LDH release. Stretching in a certain range strain range of 2%–8%, did not influence cell viability of primary tenocytes. Nevertheless, the cell death rate was 24.68% ± 4.14% in 12% stretch strain group, which significantly accelerated apoptosis of primary tenocytes (*P* < 0.05) (Fig. 3A). The LDH release was dramatically enhanced in 12% stretch strain group (*P* < 0.05) (Fig. 3B). Mkx and Tnmd mRNA expression was rapidly decreased under 12% stretch strain, 1.0 Hz frequency (*P* < 0.05, *vs.* control group, Fig. 3C). These results showed that 12% stretch strain would cause tenocytes damage, and 12% stretch strain was used in following experiment. Excessive physical forces reduced cell viability and Mkx mRNA level in vitro.

**Fig. 4:**
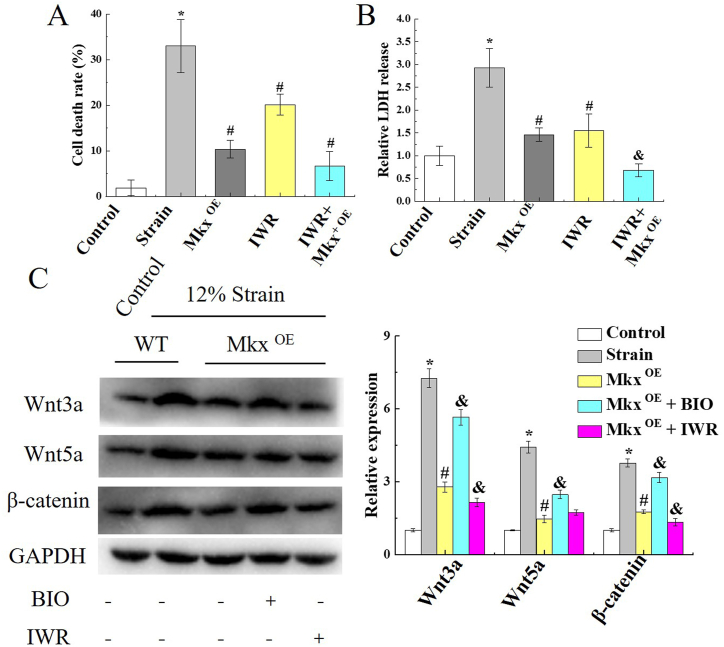
Mkx protected tenocytes from excessive stretch damage in vitro. WT, Mkx^OE^ and IWR treated tenocytes were undergone 12% stretch strain 1.0 Hz cyclic strain for 4 h, cell death of primary tenocytes was conspicuously suppressed in Mkx^OE^ tenocytes and IWR treated tenocytes as compared with WT tenocytes, by CCK8 assay (A) and LDH release (B). (C) WB of fractionated protein extracted from stretched tenocytes confirms that Wnt/β-catenin activation inhibition by Mkx and IWR. GAPDH was used as cytoplasmic controls. The full uncropped gels and blots images were shown in Fig. S1. Mean and SD are indicated (n = 5) (*, *P* < 0.05, *vs.* control; #, *P* < 0.05, *vs.* 12% strain; &, *P* < 0.05, *vs.* Mkx^OE^ strain; Tukey-Kramer post-hoc test).

To verify Mkx's protective effect against on tenocytes damage, Mkx was overexpressed in tenocytes. Then, WT, Mkx^OE^, and IWR treated tenocytes were subjected to a 12% stretch strain, cell death in primary tenocytes was significantly suppressed in Mkx^OE^ tenocytes and IWR treated tenocytes as compared with WT tenocytes (*P* < 0.05), which was 10.36% ± 1.95% and 20.15% ± 2.26%, respectively (Fig. 4A). Then the LDH release was decreased in Mkx^OE^ tenocytes and IWR treated tenocytes, and the Mkx^OE^ + IWR group had a lower level of LDH release than Mkx^OE^ tenocytes (*P* < 0.05, Fig. 4B). WB results showed that, 12% stretch strain caused high Wnt/β-catenin activation, such as Wnt3a, Wnt5a and β-catenin increased. While in Mkx-overexpressed or IWR-treated stretched tenocytes, Wnt3a, Wnt5a and β-catenin were significantly reduced (Fig. 4C). Overexpression of Mkx and treatment with IWR inhibited Wnt/β-catenin activation to reduce tenocytes damage, which is induced by excessive stretch.

**Fig. 5:**
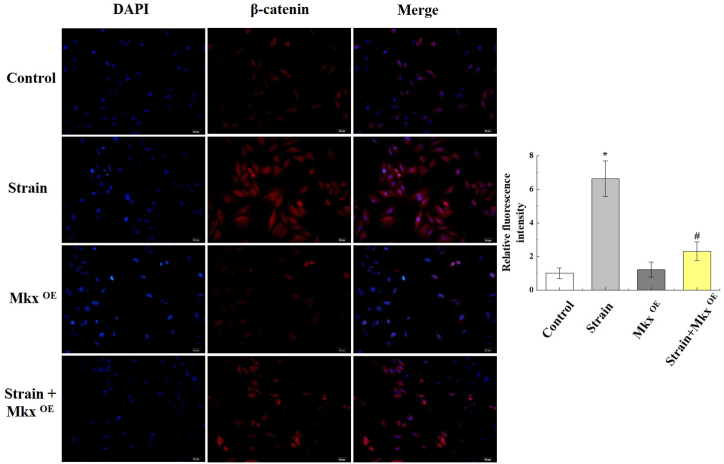
Upregulated Mkx significantly reversed the increase in β-catenin. Mkx^OE^ and WT tenocytes were undergone 12% stretch strain 1.0 Hz cyclic strain for 4 h, β-catenin expression were detected by IF after Mkx-overexpressed tenocytes. The red fluorescence intensity was analyzed statistically. Mean and SD are indicated (n = 5) (*, *P* < 0.05, *vs.* control; #, *P* < 0.05, *vs.* strain; Tukey-Kramer post-hoc test). Scale bars: 20 μm. Scale bars: 20 μm.

**Fig. 6:**
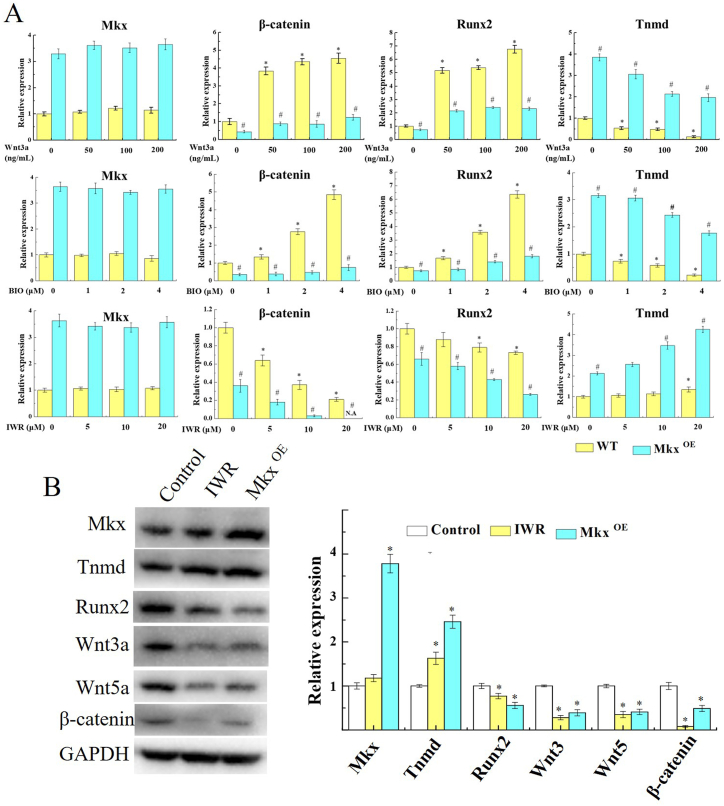
Mkx reduced Wnt/β-catenin activation in tenocytes. (A) WT and Mkx^OE^ primary mice achilles tenocytes were treated with either Wnt3a, BIO (an activator of β-catenin) or IWR (an inhibitor of β-catenin). Relative expressions of Mkx, Tnmd, Tunx2 and β-catenin in tenocytes treated with Wnt3a (0–200 ng/mL), BIO (0–2 μM), or IWR (0–20 μM) were indicated. (B) Achilles tenocytes were treated with 20 μM IWR, then Mkx, Tnmd, Tunx2, Wnt3, Wnt5 and β-catenin expression were compared with Mkx^OE^ and WT tenocytes. The full uncropped gels and blots images were shown in Fig. S1. Mean and SD are indicated (n = 5) (*, *P* < 0.05; *vs.* WT control; Tukey-Kramer post-hoc test).

**Fig. 7:**
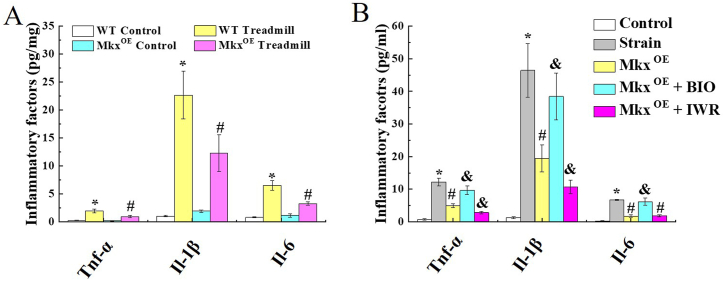
Inflammatory factors Tnf-α, Il-1β, and Il-6 levels in tenocytes. (A) Inflammatory factors Tnf-α, Il-1β, and Il-6 levels in WT and Mkx^OE^ Achilles tendon tissues, were detected by ELISA after treadmill exercise at 12 weeks. (*, P < 0.05, vs. WT control; #, P < 0.05, vs. WT treadmill). (B) ELISA analysis of Tnf-α, Il-1β, and Il-6 levels from stretched tenocytes confirms that inflammation inhibition by Mkx and IWR. Mean and SD are indicated (n = 5) (*, *P* < 0.05, *vs.* control; #, *P* < 0.05, *vs.* 12% strain; &, *P* < 0.05, *vs.* Mkx^OE^ strain). Mean and SD are indicated (n = 5).

In addition, β-catenin expression was up-regulated and detected by IF after Mkx-overexpressed tenocytes (Fig. 5). WT and Mkx^OE^ tenocytes were treated with Wnt3a (0/50/100/200 ng/mL), BIO (0/0.5/1/2 μM) or IWR (0/5/10/20 μM), qRT-PCR and WB were conducted to analysis Wnt/β-catenin pathway. Wnt3a and BIO inducedβ-catenin mRNA level in a dose-dependent manner, but IWR decreased mRNA level, and Mkx was uninfluenced both in Wnt3a, BIO and IWR, in WT tenocytes. Consistently, in Mkx^OE^ tenocytes, the ability of Wnt3a and BIO-induced β-catenin and Runx2 mRNA expression was diminished (Fig. 6A). As shown in Fig. 6B, overexpressing Mkx and treating with IWR (20 μM), Wnt3, Wnt5 and β-catenin were expressed at low levels in tenocytes. Mkx and IWR repressed Wnt/β-catenin signaling in tenocytes. Stretch strain induced β-catenin expression, while upregulated Mkx significantly reversed the increase in β-catenin. As shown in Fig. 7A, inflammatory factors Tnf-α, Il-1β, and Il-6 were significantly increased after excessinve mechanical stimulation but lower level in Mkx^OE^ treadmill group than that in WT treadmill group (*P* < 0.05).

And overexpressed Mkx or Wnt inhibitor IWR reduced inflammatory factors Tnf-α, Il-1β, and Il-6 levels but BIO could weaken the influence of Mkx overexpression (Fig. 7B).

## 4 Discussion

Tendon damage is a common clinical problem, as damaged tendon tissue heals slowly and rarely completely recuperates. However, the molecular mechanisms of how tenocytes resist physical forces to damage, are incompletely understood. We explored the functions of Mkx in mechanical damage by analyzing the Mkx overexpression mice and tenocytes. We had mainly studied the Achilles tendon, which is directly involved in interstitial spaces in the limbs [30]. Mechanical signals can sensitively cause changes in MKX expression in vivo and in vitro, with Mkx being triggered by physical exercise in Achilles tendon tissues and cellular stretching in primary achilles tenocytes of mice. Gentle stimulation could promote Mkx expression, while overload would cause the Mkx decrease to tendon damage. The novel finding that Mkx and tendon-associated genes were reduced by excessive mechanical exercise, and the decrease in tendon tension strength was evident. Mkx overexpression in Achilles tendon of mice improved the negative effects of mechanical stimulation, such as, poor tension strength, high levels of Col2a1, Acan, Runx2, Alpl, and IBSP osteogenesis- and chondrogenesis-related markers, and Wnt/β-catenin activation. In vitro, primary achilles tenocytes, Mkx overexpression would protect against tenocyte damage, oppose Wnt/β-catenin activation, and inflammation induced by excessive cellular stretch. The data shed light on a mechanically-stimulated transcriptional network in which Mkx aggregates in the Achilles tendon, and the upregulation of Mkx may be a protective mechanism that limits tendon injury and regulates abnormal cell signaling related to disease.

As an essential transcription factor of tendon development, the biological function Mkx has been revealed [13,22], and also regulates orthodontic tooth movement via osteoclast induction [31]. Our work found fatigued exercise decreased Mkx expression in tendon cells and tissues. How exactly mechanical signals affect tendon damage, and how Mkx regulates tenocyte and tendon resistance to physical forces and repair, however, is not well understood. Kayama et al. demonstrate that transmission electron microscopy observed 5 weeks adequate mechanical stimulation can promote the thickening of collagen fibers and fiber density, but that is not presented in Mkx KO mice [32]. In our mouse-treadmill model, Mkx overexpression can ameliorate the deterioration of mechanical properties of the Achilles tendon caused by excessive exercise, as well as the reversing tendon-related markers expression. In mice and rats, Mkx loss is manifested by tendon hypoplasia, such as flexor extensor, tail, and patellar tendons, while maintaining collagen orientation [22,33]. Mkx deficiency decreases tensile strength of patellar tendon in rats [33], but cell density increased in Achilles tendon of Mkx KO mice [22]. We found moderate stretching increased Mkx and tendon-associated genes in tenocytes, but increased stretching percentage resulted in a decrease in Mkx and an increase in damage. Some findings indicate that short periods of treadmill exercise can reduce the expression of tendon markers, appropriate mechanical loads may help reduce tendon stiffness and increase collagen renewal in older mice, as they have the potential to induce anabolic changes in tenocytes [34], but high-intensity treadmill exercise can also inhibit the increase of tendon markers. While our optimal stretching conditions differ from previous studies, this may be due to differences between species and different equipment used, but excessive cell stretching decreased Mkx, Col1a1, Tnmd, Fmod, and Dcn expression, with results similar to those in the previous studies [32,35]. Tendine-related markers, such as Col1a1, Tnmd, Fmod, Dcn, and Tnxb, are associated with cross-linking between collagen fibers, and their reduction may prevent normal cross-linking formation despite mechanical stimulation [36,37]. Mkx deficiency induced osteogenic and chondrogenic associated genes Col2a1, Acan, Runx2, Alpl, and IBSP can explain apparent resistance to tendon tension strength in Mkx^−/−^ mice [33]. Mkx may regulate tendons elasticity through affecting tendon cell density, cell number, ECM compounds and fiber bundles. Mkx deficiency aggravates tendon damage during excessive mechanical stimulation.

Intense exercise and extreme stretching have been reported to elevate osteogenesis- and chondrogenesis-related markers' expression levels in tendons [38,39], and our findings also confirmed this. Physiological load affects tendon stiffness, compared with cyclic load, random amplitude-modulated stretching is more likely to induce microdamage and reduce stiffness, but does not affect tenocyte metabolism [40]. Basic features of mechanical load, such as strength, frequency, time, etc., are part of the delicate balance of tendon homeostasis that regulates the phenotypes of tendon cells, osteoblasts, chondrocytes, and adipocytes [41]. Our results imply tendons and tenocytes are sensitive to amplitude and duration of mechanical stimulation. Most scholars believe that excessive tension loading causes tendon fiber injury, which is the leading cause of tendon disease. Furthermore, we found Wnt signaling pathway was abnormally activated in tendons and tenocytes that underwent excessive mechanical stimulation. The Wnt signaling pathway plays an important role in pathological calcification. Wnt signaling dysregulation has been reported in many histopathologies, such as cardiovascular calcification [42] and ossification and tissue degeneration of skin calcification [43]. Wnt signaling interacts with the bone morphogenetic protein signaling during embryonic development and bone homeostasis [44]. Liu et al. confirmed uniaxial mechanical tension induces the osteogenic differentiation of rat tendon-derived stem cells (rTDSCs) via the Wnt5a/Wnt5b/JNK pathway and which may influence the heterotopic ossification of tendon tissue subjected to excessive tension [45]. Wnt3a, β-catenin, Lrp5, and Tcf1 are expressed in chondrocyte like cells and ossified deposits in animal models and some clinical samples of tendinopathy, which are associated with failure of ossified tendon healing; In addition, Wnt3a increased the expression of ALP activity, calcium nodule formation, and osteogenic markers in rTDSCs [46]. TDSCs is a precursor of tenocytes that can differentiate into multiple lineages and play a key role in the regeneration and repair of tendon injury, while TDSCs are prone to osteogenic differentiation under excessive tension [47].

Wnt signaling is known to be activated during the repair of fractured bone and mediates bone regeneration and pathological calcification [48]. Abundant studies have shown that osteoblast maturation and calcification are reduced by inhibiting the Wnt signaling [[49], [50], [51]]. Inflammation regulates bone metabolism with consequent bone loss and enhanced fracture risk [52], and Wnt/β-Catenin-mediated inflammation is involved in new bone formation and bone loss [53]. The heterotopic ossification of tissue is observed in both the ossified failed healing animal model and clinical samples of tendinopathy. Reducing the degree of ossification in tendons through Wnt signal will benefit the protection of tendon damage. Promethazine inhibits Wnt/β-catenin signaling and improves the histological abnormalities of healing tendons, but Wnt/β-catenin inhibitor IWR-1 compromises the biomechanical properties of tendon healing [54]. Previous studies have shown that mechanical signals are essential for the formation of collagen fibers and collagen crosslinking. Overexpression of Mkx has been shown to promote tendon-related gene expression and to repress gene expression characteristic of other cell lineages [20,55], and osteogenic and chondrogenic differentiation occurs more readily in TDSCs from an Mkx^−/−^ background than in those from an Mkx^+/+^ background [33]. Mkx induces a cell state to respond to the external environment, so the tissue-resident cells and tissue itself are able to withstand and adjust to the demands of their environment. However, Mkx and its downstream origins are expressed due to load-sensitive and complex network of mechanosensitive regulators that determine whether to maintain or promote osteogenesis, and how Mkx and Wnt interact in this complex regulatory system remains to be clarified. Milet et al. show that Wnt/β-catenin signaling suppresses expressions of Scx, Mkx, and Tnmd in tendon-derived cells [56]. But Mechakra et al. demonstrate that Mkx binds to the MyoD promoter and which is the basis of global regulatory processes related to angiogenesis and Wnt signaling [20]. We found Acan, Runx2, Alpl, Wnt3, and Wnt5, and inflammatory factors Tnf-α, Il-1β, and Il-6 were significantly lower in expression in Mkx^OE^ tenocytes and tendon tissues, that suffered from excessive mechanical stimulation, when compared with WT tenocytes and tendon tissues. Furthermore, overexpressed Mkx or Wnt inhibitor IWR could reduce Wnt signal pathway, inflammatory factors Tnf-α, Il-1β, and Il-6, and osteogenesis-related genes, Wnt3a and BIO had weak capacity to reverse this regulation of Mkx in Mkx-overexpressed tenocytes but did not affect Mkx expression. As a transcription factor, mechanical forces are focused on the induction of Mkx, but the physiological role of Mkx in vulnerable conditions is not fully explored.

The involvement of TGF-β, mTORC1, and the Wnt pathway in tendinopathy has been established [46,57,58]. The regulation of Mkx gene networks may have important therapeutic effects, because tendinopathy is a common disease, which is difficult to completely cure. Progress in tendon repair and bioartificial tissue has been slow due to a lack of understanding of molecules. But this study showed that Mkx played an important role in Wnt signaling in tendon injury, which may help in the treatment of tendinopathy and determine optimal training conditions or post-injury rehabilitation programs for athletes to promote effective tendon healing. Mkx may be stimulated by mechanical stretch as a mechanosensor, binding to Wnt3a or Wnt5a to repress osteogenesis and calcification in tendon tissues. Yet, excessive stimulus will break this balance to induce tendon damage and tendinopathy. Therefore, linking mechanical forces to the Mkx-directed gene program is essential for organized tendon repair and protection.

## 5 Conclusions

Based on our results, we conclude that excessive stimulus can promote tendon damage, and the Mkx/Wnt/β-catenin pathway is involved in this regulatory process. This study sheds light on the effects of Mkx/Wnt/β-catenin on tendon damage and will ultimately contribute to an understanding of the mechanism that underlies the advanced Achilles tendon injury induced by excessive mechanical loading, which may be a potential therapeutic target for tendon repair and regeneration.

## Ethics approval and consent to participate

All animal experiments were conducted in accordance with the program approved by the Institutional Animal Care and Use Committee at the Bijie Traditional Chinese Medicine Hospital (NO. 22052501).

## Funding

This study was supported by a grant from the 10.13039/501100004826Natural Science Foundation of Beijing (No. 7232211), 10.13039/501100018555Science and Technology Support Plan of Guizhou Province ([2023] general 088), Science and Technology Research Topic of Traditional Chinese Medicine and Ethnic Medicine in Guizhou Province (QZYY-2023-022 and QZYY-2023-025), and 10.13039/501100017596Natural Science Basic Research Program of Shaanxi (NO·2024SF-YBXM-217).

## Data availability statement

The raw data supporting the conclusions of this manuscript will be made available by the authors, without undue reservation, to any qualified researcher.

## CRediT authorship contribution statement

**Ziming Liu:** Writing – original draft, Visualization, Conceptualization. **Wenfeng Han:** Writing – original draft, Conceptualization. **Jiao Meng:** Formal analysis. **Yanbing Pi:** Methodology, Data curation. **Tong Wu:** Methodology, Data curation. **Yifei Fan:** Methodology, Data curation. **Qinwei Guo:** Methodology, Data curation. **Xiaoqing Hu:** Methodology, Data curation. **Yuhua Chen:** Funding acquisition, Formal analysis. **Wenxiao Jiang:** Writing – review & editing, Conceptualization. **Feng Zhao:** Writing – review & editing, Visualization, Supervision, Funding acquisition, Conceptualization.

## Declaration of competing interest

The authors declare that they have no known competing financial interests or personal relationships that could have appeared to influence the work reported in this paper.
