# Supplementary figures and images for: Mohawk protects against tendon damage via suppressing Wnt/β-catenin pathway
Source: Heliyon. 2024 Feb 6;10(4):e25658. doi: 10.1016/j.heliyon.2024.e25658 (PMC10867664; doi:10.1016/j.heliyon.2024.e25658)

**Figure S1 The full uncropped gels and blots images.**

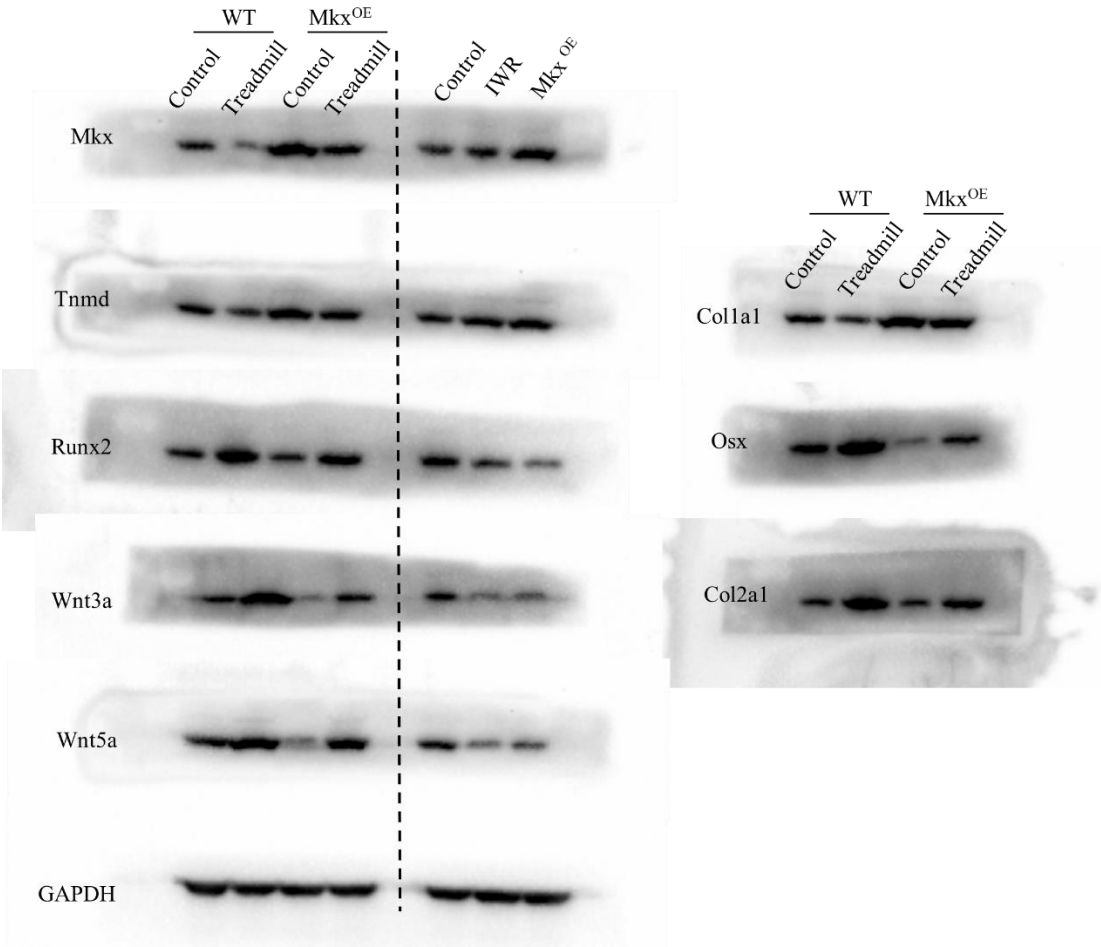

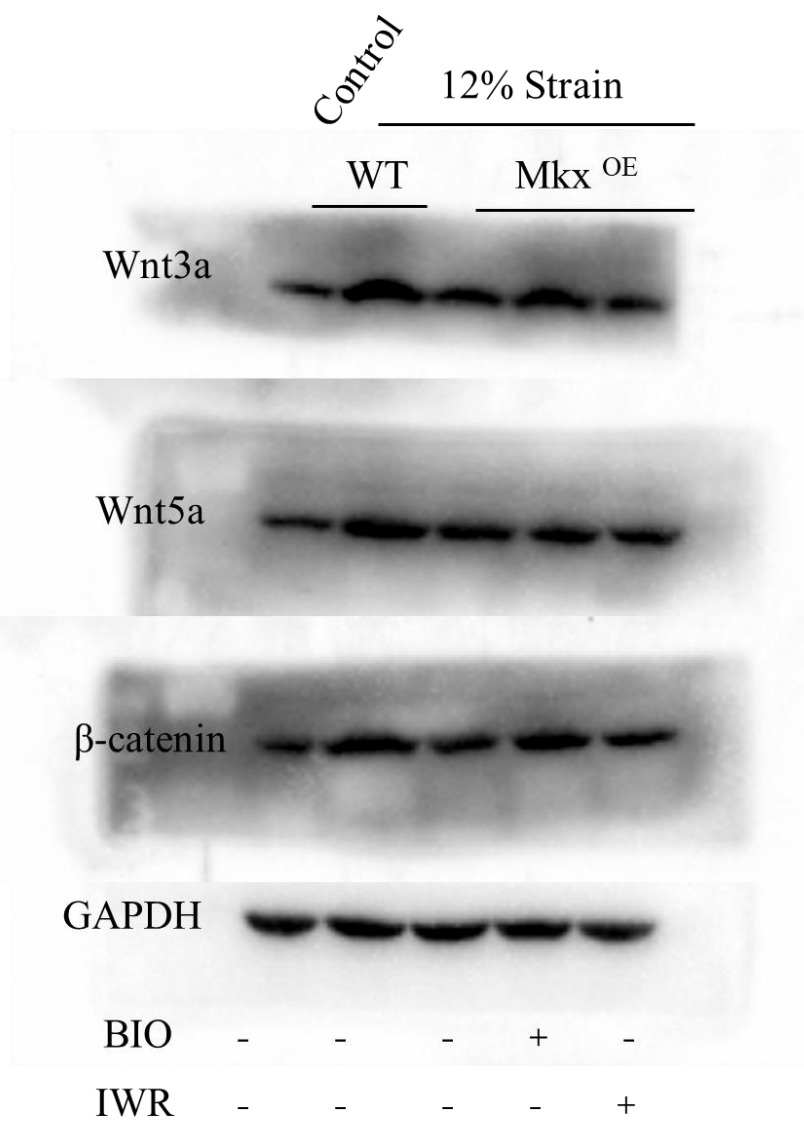

Supplement: Multimedia component 1 [file mmc1.pdf]
